# Digital Rectal Examination and Balloon Expulsion Test in the Study of Defecatory Disorders: Are They Suitable as Screening or Excluding Tests?

**DOI:** 10.1155/2016/8654314

**Published:** 2016-10-26

**Authors:** Ana C. Caetano, André Santa-Cruz, Carla Rolanda

**Affiliations:** ^1^Department of Gastroenterology, Braga Hospital, Braga, Portugal; ^2^Life and Health Sciences Research Institute (ICVS), School of Health Sciences, University of Minho, Braga, Portugal; ^3^ICVS/3B's-PT Government Associate Laboratory, Guimarães, Braga, Portugal; ^4^Department of Internal Medicine, Braga Hospital, Braga, Portugal

## Abstract

*Background*. Rome III criteria add physiological criteria to symptom-based criteria of chronic constipation (CC) for the diagnosis of defecatory disorders (DD). However, a gold-standard test is still lacking and physiological examination is expensive and time-consuming.* Aim*. Evaluate the usefulness of two low-cost tests—digital rectal examination (DRE) and balloon expulsion test (BET)—as screening or excluding tests of DD.* Methods*. We performed a systematic search in PUBMED and MEDLINE. We selected studies where constipated patients were evaluated by DRE or BET. Heterogeneity was assessed and random effect models were used to calculate the sensitivity, specificity, and negative predictive value (NPV) of the DRE and the BET.* Results*. Thirteen studies evaluating BET and four studies evaluating DRE (2329 patients) were selected. High heterogeneity (*I* ^2^ > 80%) among studies was demonstrated. The studies evaluating the BET showed a sensitivity and specificity of 67% and 80%, respectively. Regarding the DRE, a sensitivity of 80% and specificity of 84% were calculated. NPV of 72% for the BET and NPV of 64% for the DRE were estimated. The sensitivity and specificity were similar when we restrict the analysis to studies using Rome criteria to define CC. The BET seems to perform better when a cut-off time of 2 minutes is used and when it is compared with a combination of physiological tests. Considering the DRE, strict criteria seem to improve the sensitivity but not the specificity of the test.* Conclusion*. Neither of the low-cost tests seems suitable for screening or excluding DD.

## 1. Introduction

Defecatory disorders (DD) are common in the community with an incidence of 22 per 100,000 person years [1]. DD may result from disordered anorectal function (e.g., inadequate rectal propulsive forces and/or increased resistance to evacuation) or rectal structural disturbances (which prevent its reservoir function); these pathophysiological mechanisms may coexist [2–7]. Rome III criteria [8] provide a symptom-based definition of chronic constipation (CC) and recognize subgroups of CC based on both symptoms and physiological criteria. According to Rome III classification, the diagnosis of DD is established when a patient with CC has evidence of at least two physiological impairments in any of the anorectal tests—balloon expulsion test (BET), imaging, anorectal manometry (ARM), or electromyography (EMG). Rome III diagnostic criteria fulfilled for the last 3 months with symptom onset at least 6 months prior to diagnosis for functional defecation disorders are as follows:

1. The patient must satisfy diagnostic criteria for functional constipation.
2. During repeated attempts to defecate the patient must have at least two of the following:
  - Evidence of impaired evacuation, based on BET or imaging.
  - Inappropriate contraction of the pelvic floor muscles (i.e., anal sphincter, or puborectalis) or less than 20% relaxation of basal resting sphincter pressure by ARM, imaging, or EMG.
  - Inadequate propulsive forces assessed by ARM or imaging.

Diagnostic criteria for functional constipation are as follows:

1. The patient must include two or more of the following: (a) straining during at least 25% of defecations, (b) lumpy or hard stools at least 25% of defecations, (c) sensation of incomplete evacuation at least 25% of defecations, (d) sensation of anorectal obstruction/blockage at least 25% of defecations, (e) manual maneuvers to facilitate at least 25% of defecations (e.g., digital evacuation, support of the pelvic floor), and (f) fewer than three defecations per week.
2. Loose stools are rarely present without the use of laxatives.
3. There are insufficient criteria for irritable bowel syndrome.

Based on evidence that shows substantial symptom overlap among the different pathophysiological entities of CC [9–11], Rome III criteria state the need of anorectal tests to substantiate the diagnosis of DD. An extensive technical description of these tests is made elsewhere [6, 12].

However, the findings on these different tests may not be in agreement [11, 13]. Thirty percent of patients with marked evacuatory symptoms have negative tests for DD [11] and results compatible with DD are documented in around 25% of healthy individuals [14–18]. Most authors from tertiary centres emphasize the phenotypic heterogeneity of DD [19, 20] and the unhelpful settings in which these tests are performed [21] to explain this disagreement. However, they tend to trust the potential benefit of an extensive and ultimately enhanced diagnostic approach [7, 22]. Another aspect to take into account is the cost involved in diagnosing DD. The exact impact of CC diagnostic assessment in Western Europe healthcare systems is unknown [23] but an American study by Rantis Jr. et al. [24] reports a costly work-up without even considering the indirect cost [25].

A low-cost screening option to identify DD in clinical settings with limited resources would have clinical [26] and financial advantages.

The digital rectal examination (DRE) is the simplest and cheapest clinical tool for identifying DD, thoroughly described by Talley [27]. Tantiphlachiva et al. [28] proposed that DD could be diagnosed by DRE, if two of the following criteria were present: (1) a paradoxical anal contraction or impaired anal relaxation, (2) impaired push effort, and (3) absence of perineal descent. Orkin et al. [29] compared a semiquantitative DRE scoring system (DRESS) and found it reasonably accurate in comparison to ARM for assessing anal resting tone and squeeze function and for identifying dyssynergia. Due to the emphasis given nowadays to technology rather than to clinical skills [30], this simple tool may sometimes be underused [29].

The BET is a simple procedure, first described by Preston and Lennard-Jones [2], that evaluates a patient's ability to evacuate a filled balloon. Different methodologies consider air filled or water-filled balloon and the lying or seated position to perform the BET. Recommended time values range from less than 1 minute to up to 5 minutes [31]. Recent studies showed that the inability to expel the balloon is suggestive of DD [16, 32]. Interestingly, Minguez et al. [33] proposed that a normal test would exclude DD. Contradictory data showed that some patients with DD could expel the balloon [11, 19, 34] making this test alone apparently insufficient to exclude a diagnosis of DD.

Chiarioni et al. [34] addressed the issue of BET reproducibility and found a perfect reproducibility in 280 patients with constipation (98%, cut-off < 2 minutes), adding value to this attractive screening option.

Our aim was to conduct a systematic review of the studies that included patients with CC who performed a low-cost anorectal evaluation (DRE or BET) to determine the sensitivity and specificity of these tests for the diagnosis of DD and their potential value as screening or excluding tests.

## 2. Method

We conducted a literature search in the online databases MEDLINE and PUBMED. The search terms included chronic constipation, functional constipation, defecatory disorders, dyssynergic defecation, dyssynergic evacuation, pelvic floor dysfunction, pelvic floor dyssynergia, balloon expulsion test, balloon evacuation, digital rectal examination, and digital rectal exploration. Besides, a manual search of the references from previously published systematic reviews was also performed to identify additional studies of interest. The search was limited to manuscripts published in English between 1975 and 2015 in adult population. Abstracts were screened and potentially relevant articles were reviewed.

### 2.1. Selection of Studies and Data Extraction

We selected studies where constipated patients were evaluated by DRE or BET. The definition of constipation is not clearly defined in most articles so we considered any study evaluating patients with constipation. For analysis we included only the studies in which (i) patients were unselected so they did not have a defined subtype of constipation, (ii) criteria for DD were clearly stated and comparative physiological test was defined by the author, and (iii) the data of the manuscript was sufficient to determine sensitivity, specificity, and NPV for the DRE or the BET. Manuscripts with a study population with less than 25 patients were excluded.

Caetano and Rolanda extracted the data. The inclusion criteria and the extracted data are presented in Table 1.

### 2.2. Meta-Analysis and Subgroup Analyses

The sensitivity, specificity, and NPV of the BET and the DRE were calculated for the included studies and presented in forest plots. Random effects models were used to provide pooled estimates of sensitivity and specificity and their corresponding 95% confidence intervals (CI). Statistical heterogeneity among the studies was investigated using *I* ^2^ statistic. All analyses were performed using the Comprehensive Meta-Analysis version 3 (Biostat, Englewood, NJ, USA).

## 3. Results

### 3.1. Eligible Studies

**Figure 1:**
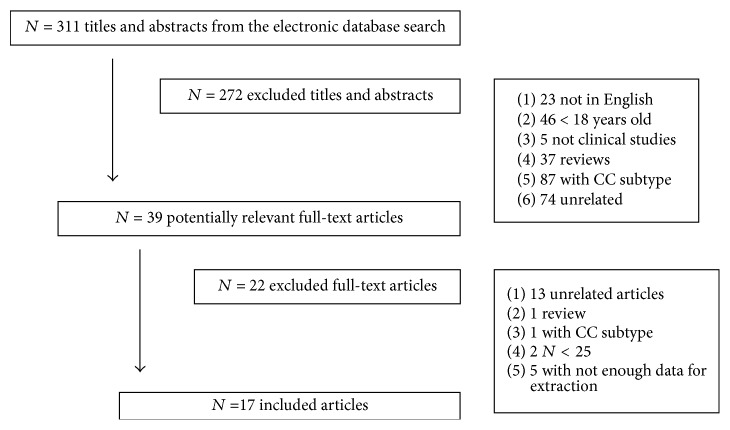
Flowchart of the literature search and study selection process.

With the electronic database and manual search, a total of 311 titles and abstracts were screened and 39 full-text manuscripts were reviewed. Seventeen articles fulfilled the criteria and were included for the analysis. We present the graphic flow in Figure 1. The included articles were published from 1987 until 2015. We used data regarding the BET in 13 studies and data regarding the DRE in 4 studies. The clinical series varied between 32 and 295 patients completing a total of 2329 patients. The data from the majority of studies (88%) were collected prospectively. Rome criteria for CC were used in 7 studies. The combined mean age of the patients was 47,5 years and 75% were women (excluding 3 studies that did not describe gender distribution).

### 3.2. Balloon Expulsion Test

Thirteen studies met our inclusion criteria and supplied sufficient data to determine sensitivity, specificity, and NPV for the BET.

**Figure 2:**
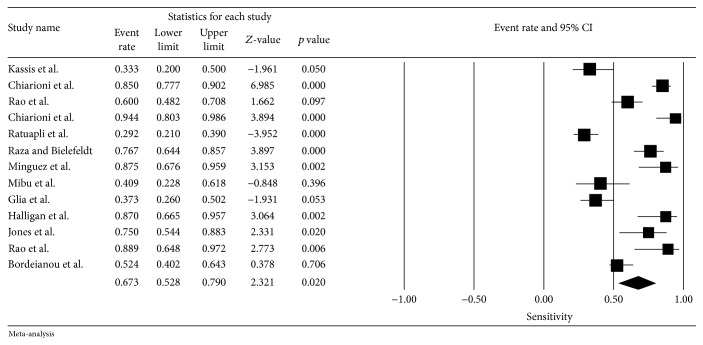
Summary measures for sensitivity of BET analysis.

**Figure 3:**
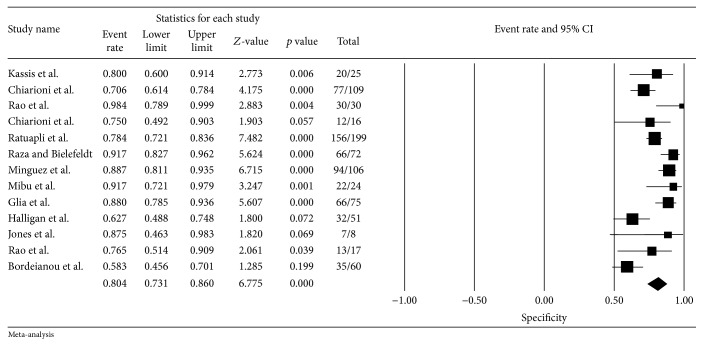
Summary measures for specificity of BET analysis.

The meta-analysis showed high heterogeneity (*I* ^2^ = 90%) among these 13 studies. Pooled sensitivity (95% CI) for failed or impaired balloon expulsion by any criteria was 67% (CI 53–79%) and pooled specificity was 80% (CI 73–86%). The sensitivity and the specificity did not change when we limited the analysis to the studies with Rome criteria for CC (5 studies, sensitivity of 65% and specificity of 78%).  Table 2 summarizes the data related to the BET and Figures 2 and 3 show the forest plot for the sensitivity and the specificity of the BET.

Taking into consideration the characteristics of the BET, when we evaluate the studies that used only water as simulated stool (10 studies), sensitivity was 71% and specificity was 80%. In the evaluation of studies that used a volume ≥ 50 mL or the seated position (11 and 12 studies, resp.), the sensitivity was 67% and the specificity was 80%. When we performed the subanalysis of the BET with normal time to evacuate ≤ 2 minutes (4 studies), sensitivity rose to 74% (CI 47–90%) and the specificity rose to 84% (CI 71–92%). The subanalysis of the gold-standard comparative test showed sensitivity and specificity of the BET of 72% and 81% in comparison to ARM (5 studies), 53% and 77% in comparison to defecography (4 studies), and 88% and 85% in comparison to a combination of tests (2 studies [33, 35]). The pooled NPV of the BET was 72% (CI 63–80%).

### 3.3. Digital Rectal Examination

Four studies considering the DRE were included after careful evaluation. Once more, high heterogeneity between studies was established by the meta-analysis (*I* ^2^ = 91%). The pooled sensitivity (95% CI) for the diagnosis of DD with the DRE using any criteria was 80% (CI 64–90%) and the pooled specificity was 84% (CI 64–94%).  Table 3 reviews the data regarding the DRE.

When we consider only studies using Rome criteria for CC [28, 36], the sensitivity was 78% and the specificity was 90%. When we repeated the analysis with studies with precise DRE diagnostic criteria for DD [28, 37], the sensitivity was 86% and the specificity was 72%.

The pooled NPV of the DRE was 64% (CI 37–85%).

## 4. Discussion

Nowadays, when medicine faces new challenges concerning cost-effectiveness, a cheaper but satisfactory diagnostic approach of DD that promotes an adequate selection of complementary tests and an earlier and targeted treatment seems ideal. This article intends to follow a creative approach to the subject “diagnosis of the DD” with the low-cost tests evaluated as screening or excluding options in patients with CC. With this idea in mind, we were able to systematically gather a vast collection of data related to the BET and the DRE.

Previous reviews [22, 38] collected important data related to all anorectal physiological tests and faced diverse challenges: variable diagnostic criteria, different protocols of physiological tests, and lack of definitions for positive results. As expressed in Tables 2 and 3, we also faced heterogeneous protocols and variable diagnostic criteria in each centre; on the other hand, we recognize an increased effort to define negative and positive results.

Considering the data from the 17 selected articles, this meta-analysis evaluated a total of 2329 patients and calculated a pooled sensitivity and specificity for the BET of 67% and 80%, respectively, and a pooled sensitivity and specificity for the DRE of 80% and 84%, respectively.

As the BET technique was not standardized, the included articles described different methodologies [33, 34, 39–43]. Most studies of this meta-analysis described the use of the seated position (12 studies) probably because this method better resembles the act of defecation. Excluding the studies that filled the balloon with air [13, 41, 42], the sensitivity and the specificity of the water-filled BET did not change. Studies that evaluated different sizes and consistencies of simulated stools found that small and hard stools are harder to expel than large softer stools [17, 44]. Although probably air simulates inadequately the stool weight and consistency, we did not see any difference in sensitivity and specificity, perhaps because of the low number of patients evaluated (301 out of 1450). Another aspect to take into account is the volume of the balloon—the majority of the studies reported fixed volumes in their series but the volume described varied between 10 mL [45] and 60 mL [43]; in addition, a study described a variable volume (median volume of 250 mL) related to the permanent desire to evacuate [33]. When we use the cut-off of ≥50 mL, the sensitivity and specificity did not change, nor when we exclude only Minguez series (median volume of 250 mL). As far as we know, no study compared fixed and variable volumes on the testing protocol of the BET.

Also most studies do not report the balloon material—silicone or latex—that also influences the volume associated with first sensation, urgency, and maximum discomfort [31]. Perhaps with a larger number of patients and a prospective and controlled comparison between different volumes and materials, a more correct performance of the BET in terms of sensitivity and specificity is possible. The time given to the patient to evacuate the balloon also varied between 1 [33] and 5 minutes [5, 13, 35]. When we use the cut-off of 2 minutes as the limit between normal and abnormal BET as proposed by Chiarioni et al. [34], the pooled sensitivity and specificity rose to 74% and 84%, respectively.

The majority of the studies reviewed in this meta-analysis were anterior to 2014 and did not define the cut-off time for normal/abnormal BET or use higher cut-off values. Probably the use of a consistent and low cut-off time for the BET in future studies will improve the value of the test. Due to all these issues, studies that use a standardized methodology of the BET, including balloon volume, are necessary. Standardization of the BET is relevant not only to diagnosis but also to treatment [46].

The analysis of the comparative anorectal test showed us that the pooled sensitivity and specificity of the BET is equivalent to ARM, but it is lower than the pooled sensitivity and specificity of the defecography. It is higher when compared to the pooled sensitivity and specificity of the combination of the tests. The consistency of results in the subanalysis of ARM is easily explained by the fact that both the BET and the ARM are purely physiological tests while defecography has also a morphological component assessment which makes it not so suitable to use as comparative test. Only two studies [33, 35] used a combination of tests to diagnose DD which may underestimate the prevalence of dyssynergia in their study population. However considering that Rome III criteria also recommend at least two morphological or physiological abnormalities to diagnose DD (in one or more tests), the BET performance comparing to a combination of tests is probably more reliable. Although we described a range of possible explanations for the moderate sensitivity and specificity of the BET compared to the “classic” physiological tests, the large overlap between normal subjects and patients with symptoms may also be explained by the BET being an unphysiological maneuver.

Although guidelines highlight the importance of rectal examination for identifying DD [6], we only collected 4 studies evaluating DRE that met our inclusion criteria. The pooled sensitivity and specificity were 80 and 84%, respectively. The small number of studies along with the heterogeneity revealed by the meta-analysis prevents us from drawing any conclusion from these data. When we evaluate only the studies that use Rome criteria [28, 36], the sensitivity and the specificity were comparable. None of the selected studies used the DRESS published by Orkin et al. in 2010 [29]. Tantiphlachiva et al. [28] and Soh et al. [37] described their own diagnostic criteria for DD using DRE, while two earlier studies [36, 47] used expert self-reported evaluation. When we analyse only the studies with more strict criteria, the sensitivity rose to 86% and the specificity decreased to 72%. Although we cannot take any definitive conclusion from this small number of patients, a possible explanation is that even less skilled practitioners can be aware of the possible abnormalities in anorectal examination when more descriptive and explanatory criteria are used. When strict criteria are used, the decreased specificity is probably related to the exclusion of knowledge of the experts in the diagnosis of DD using the DRE. We cannot deny the subjectivity implied in a physical examination—the question is if we can use it with sufficient know-how in benefit of the patient. Probably with more studies, eventual validation, and widespread use of the DRESS or Tantiphlachiva criteria, we will be capable of understanding the exact role of the DRE in diagnosing DD. It was not possible to run a subanalysis based on the comparative anorectal test because all studies except one [37] used a combination of symptoms and/or exams.

The NPV of the BET and the DRE was 72% and 64%, making these tests unsuitable for excluding the diagnosis of DD in constipated patients.

This meta-analysis has several limitations. There was no enough consistency among studies, which influenced our estimated results. The criteria to define CC were variable, from not defined or self-reported to Rome III criteria. The lack of standardization of the BET protocols made the comparison of studies problematic. Also the absence of a true comparative gold-standard test or unique diagnostic criteria for DD makes any review on the subject a difficult task. At last, another limitation, inherent to all anorectal physiological tests, is the imperfect simulation of the act of defecation due to the influence of laboratory conditions.

In conclusion, with current data, although the importance of the BET and the DRE in the investigation of constipated patients is obvious, so far we cannot use these low-cost tests with a screening or exclusion purpose. There is a need for prospective studies with descriptive and consistent methodology to evaluate its utility and cost-effectiveness and confirm its reproducibility in DD diagnostic diagram. For now, the results presented in this manuscript demonstrate that the BET and the DRE should be interpreted alongside the results of the other tests of anorectal function.

## Figures and Tables

**Table 1:** Inclusion criteria and extracted data.

Studies	*N* > 25 Patients with chronic constipation (no strict criteria) Patients with no specified subtype of chronic constipation Performance of DRE or BET (in comparison to a defined physiological test) Sufficient data to calculate sensitivity, specificity, NPV

Data	Gender distribution Mean age DRE and BET characteristics Criteria for positivity in DRE and BET Comparative anorectal physiological test Criteria for diagnosis of defecatory disorder in physiological test Number of true positive, true negative, false positive, false negative

DRE: digital rectal examination. BET: balloon expulsion test.

**Table 2:** Summary of studies with balloon expulsion test.

Study reference	*N*	Gender (fem)	Age (y)	BET (simulated stool, volume (mL), body position, time (min))	Comparative test (used criteria)	Se	Sp	PPV	NPV
[39]	61	61	50	W 50 S 2	HRM and/or DEF pr-pc or pr-nr, ce-i	33	80	71	45
[34]	236	264	44	W 50 S 2	ARM pr-pc or pr-nr	85	71	77	80
[11]	100	80	53	W 50 S 3	ARM pr-pc	60	1	100	52
[5]	52	49	35	W 50 S 5	ARM pr-pc or pr-nr	94	75	89	86
[40]	295	295	48	W 50 S 3	HRM pr-pc or pr-nr	29	78	39	70
[41]	132	256	52	A 50 S 2	ARM pr-pc or pr-nr	76	92	92	80
[33]	130	124	38	W SD S 1	ARM + DEF pr-pc or pr-nr, ce-i	88	89	64	97
[42]	46	34	46	A 20 S	DEF ce-i	41	92	82	63
[43]	134	112	52	W 60 S	DEF ce-i or ce-f	37	88	71	64
[45]	74	65		W 10	DEF ce-i or ce-f	87	63	51	91
[14]	32		46	W 50	EMG i	75	88	95	54
[35]	35	30	44	W 50 S 5	ARM + DEF/CTT pr-pc or pr-nr, ce-i, stt	89	76	80	87
[13]	123	118	51	A 60 S 5	DEF ce-i	52	58	57	54

BET: balloon expulsion test, HRM: high resolution manometry, ARM: anorectal manometry, DEF: defecography, EMG: electromyography, and CCT: colonic transit time.

BET: W: water, A: air, SD: sustained desire to evacuate, and S: seated position.

HRM or ARM: pr-pc (puborectalis paradoxical contraction) or pr-nr (puborectalis nonrelaxing).

DEF: ce-i (contrast evacuation impaired) or ce-f (contrast evacuation failed in 30 s).

EMG: i (activity increased).

CTT: stt (slow transit time).

**Table 3:** Summary of studies with digital rectal examination.

Study reference	*N*	Gender (fem)	Age (y)	DRE (used criteria)	Comparative test (used criteria)	Se	Sp	PPV	NPV
[37]	268	152	64	2 of as-pc/as-nr, pe-i, pd-a	HRM (type I–IV DD)	93	59	91	66
[28]	209	18	41	2 of as-pc/as-nr, am-nc, pd-a	ARM + BET or CTT pr-p or pr-nr, ne, stt	73	85	97	31
[36]	168		44	pr-pc	ARM + CTT	83	95	98	65
[47]	136			pr-pc	DEF + EMG aa-ni, i	58	88	62	87

DRE: digital rectal examination, HRM: high resolution manometry, ARM: anorectal manometry, DEF: defecography, EMG: electromyography, and CCT: colonic transit time.

DRE: as-pc (anal sphincter paradoxical contraction), as-nr (anal sphincter nonrelaxing), am-nc (abdominal muscles not contracted), pd-a (perineal descent absent), and pe-i (push effort impaired).

HRM or ARM: pr-pc (puborectalis paradoxical contraction) or pr-nr (puborectalis nonrelaxing).

DEF: aa-ni (anorectal angle not increased).

EMG: i (activity increased).

CTT: stt (slow transit time).

BET: ne (not expelling a 50 mL water balloon in 1 minute).
